# Animal bite injuries in the accident and emergency unit at Mulago Hospital in Kampala, Uganda

**DOI:** 10.11604/pamj.2019.33.112.16624

**Published:** 2019-06-13

**Authors:** Robert Wangoda, Jane Nakibuuka, Edith Nyangoma, Samuel Kizito, Teddy Angida

**Affiliations:** 1 Department of Surgery Masaka Regional Referral Hospital P.O. Box 18, Masaka, Uganda; 2 Department of Medicine Mulago National Referral Hospital P. O. Box 7051, Kampala, Uganda; 3 Clinical Epidemiology Unit, Makerere University College of Health Sciences P.O. Box 7051, Kampala, Uganda; 4 Statistics Unit Mulago National Referral Hospital P.O. Box 7051, Kampala, Uganda

**Keywords:** Animal bite, injuries, Mulago, Uganda

## Abstract

**Introduction:**

Animal bite injuries are a common public health concern in Uganda. We sought to characterize animal bite injuries among patients presenting to Mulago National Referral Hospital in Kampala, Uganda.

**Methods:**

This was a cross sectional study from 1^st^ September to 30^th^ November 2011. Participants were animal bite injury victims presenting to the accident and emergency (A&E) unit at Mulago hospital and were consecutively enrolled into the study. Socio-demographics, severity and patterns of injury, health seeking and dog handling behaviours were assessed using a standardized questionnaire. Descriptive statistics was used to summarize participant characteristics and the animal bite injuries. Poisson regression model's incident rate ratios (IRR) was used to explore the relationship of the number of days to accessing treatment at Mulago hospital with; a) received prior first aid, b) animal bite injury sustained during day time, c) unknown dog and d) victim resident in Kampala. Data were analyzed using STATA version 12.0 and statistical significance set at P < 0.05.

**Results:**

Of 25,420 patients that presented to the A&E unit during the study period, 207 (0.8%) had animal bite injuries, mean age 22.7 years (SD 14.3), 64.7% male, and 40.1% were <18 years. Majority 199 (96.1%) were bitten by a lone unrestrained and un-signaled dog that had bitten someone else in 22.2% of cases, and eight victims (0.4%) were attacked in canine gangs of 2-5 dogs. Rabies vaccination was confirmed in only 23 dogs (11.1%) as 109 (52.7%) were unknown to the victims or the communities. One hundred and eighteen victims (57.0%) sustained the dog bites within Kampala district whilst the rest occurred near or far from Kampala district, and the victims especially referred to access anti-rabies vaccine. Of 207, 189 victims (91.3%) presented within 2.6 days (SD ± 4.3). Two hundred victims (96.6%) sustained extremity injuries while the rest had injuries to other body parts. All injuries were minor and managed on out-patient basis with wound dressing, analgesics, prophylactic antibiotics and anti-rabies vaccination. Victims who received prior first aid had a rate of 1.7 times greater for seeking treatment at Mulago hospital (IRR 1.7, 95% CI 1.4-2.1) compared to those that had no prior first aid. Participants who sustained the animal bite injuries during day time had a rate of 1.6 times greater for seeking treatment at Mulago hospital (IRR 1.6, 95% CI 1.3-2.1) compared to those that sustained injuries at other times. Participants bitten by unknown dog and participants residing in Kampala had IRR 0.7, 95% CI 0.5-0.9 and IRR 0.6, 95% CI 0.5-0.8 respectively of accessing treatment at Mulago hospital compared to bitten by known dog and not residing in Kampala.

**Conclusion:**

Dog bites injuries from unrestrained, un-signaled dogs are the commonest source of animal bite injuries especially among children (<18 years). Vaccination against rabies was only confirmed for a very small number of dogs, as majority were unknown and likely stray dogs. Government and public sensitization is urgently required to limit stray dogs, vaccinate dogs and restrain them to prevent a grave probability of a looming canine rabies epidemic.

## Introduction

Animal bites in humans are a serious public health concern because bites from rabies-infected animals represent the single most important source for the transmission of rabies to humans worldwide. The biggest burden of animal bite injuries, which are predominantly from dog bites are found in Asia and Africa where an estimated 55,000 persons die from rabies each year. Other than the association with rabies, animal bites may lead to permanent disfigurement requiring reconstructive surgery, disability, infection, and in rare cases attacks may be fatal [1-3]. Animal bites also result in a large monetary expense for treatment, post exposure treatment of rabies and in some cases hospitalization and yet the burden of animal bite injuries and their consequences such as rabies are mainly felt by poor and vulnerable populations in rural communities who may not access medical services or even pay for them [1-7].

Although animal bites injuries are a common health concern in Uganda, accurate morbidity and mortality rates associated with these injuries and rabies in particular are limited. A previous study estimated an incidence of animal bites to be 39.6 bites per 100,000 people [3]. However, these estimates may be an under approximation of the magnitude of animal bite injuries in Uganda. This is because surveillance for dog and other animal bites is inadequate more so in rural and peri-urban areas where many of the cases occur and many patients may not report to health care facilities. Accident and emergency (A&E) department data have been used to describe the risk factors and public health impact of animal bites [7]. This study characterized animal bites injuries among patients presenting to the Accident and Emergency Unit at Mulago Hospital.

## Methods

### Study design and setting

This was a cross-sectional study conducted at Mulago National Referral and Teaching Hospital's Accident and Emergency Unit. The hospital has a 1,500 bed capacity and at the time of the study, Mulago Hospital's A&E unit was the only public emergency and trauma unit that served Kampala city and its neighboring towns with an estimated catchment population of two million people. The A&E unit attends to approximately 300 patients daily that seek acute care for various medical and surgical emergencies. The study was approved by Mulago Hospital's Research and Ethics Committee. Informed consent and assent for participation in the study was obtained from participants prior to enrollment.

### Recruitment

During a three-month period from 1^st^ September to 30^th^ November 2011, 25,420 patients presented to the accident and emergency unit. Of these, 207 patients that presented to the A&E unit with complaints of animal bites were identified from the daily triage A&E registers and requested to participate in the study. Following a written informed consent or assent to participate in the study, participants were consecutively enrolled into the study.

### Study procedures

An interviewer administered a pre-tested and standardized questionnaire. Information obtained included selected socio-demographic characteristics such as age, gender, district, education status, occupation. Clinical information obtained included: date and time of animal bite, what animal was involved, site of injury, activity and setting at time of bite, ownership of the animal, number of animals attacking the victim, vaccination status of the animal, health status of the animal, and victim's health seeking behavior such as initial treatment prior to presentation to the A&E, and subsequent treatment or hospitalizations. The time of bite was categorized as either day or night. Animal owner ship was categorized as home/family animal, neighbor's animal, police animal/dog, and if the victim did not know the animal's owner, it was specified as “wild/unknown”. Activity at the time the animal bite occurred was classified as playing with dog, beating dog, walking or running. Animals were labeled restrained if at the time of the bite they were chained or in a locked up in a physical space unable to get away. The animal's health status was assessed by asking if the dog was still alive and if not, whether it died of disease or killed by the community, as well as assessing how many other people had been bitten by the same animal.

### Data management and analysis

Data from the questionnaires was entered centrally into one excel spread sheet database. This was checked for errors, and a copy was frozen. Descriptive statistics of means for continuous data, frequency and percentages for categorical data were used to summarize data on socio-demographic variables and characteristics of animal bites. A poisson regression model was employed to explore the relationship of the number of days to accessing treatment at Mulago hospital with; a) received prior first aid, b) animal bite injury sustained during day time, c) unknown dog and d) victim resident in Kampala, after assessing and ruling out suitability for negative binomial model. Incidence Rate Ratio (IRR) was used as the measure of association. For all the models only factors with p-values of 0.2 or less at bivariate analysis were entered in a multivariate model. Confounding factors were assessed at a 10% difference between the measure of association for the unadjusted and adjusted models. All statistical analysis was performed using STATA software version 12.

## Results

### Socio-demographic characteristics of the study participants and prevalence of animal bite injuries

Over a period of three months from 1^st^ September to 30^th^ November 2011, 25,420 patients presented to Mulago Hospital's A&E Unit. Of these, 207 presented with animal bite injuries, representing a prevalence of 0.8%. Table 1, Table 2, Table 3, Table 4 show results of univariate and multivariate data analysis respectively. The mean age of the victims was 22.7 years (SD 14.3), 134 (64.7%) were male and 83 (40.1%) were aged < 18 years. One hundred and eighteen victims (60.8%) sustained the animal bites within Kampala district whilst the rest occurred outside of Kampala district and the victims referred to Mulago hospital to access anti-rabies vaccine. Notably, one patient travelled from as far as Rukungiri district which is 300 kilometers from Kampala district to access post exposure prophylaxis with anti-rabies vaccine. The socio-demographic characteristics of the study participants are as shown in Table 1.

**Table 1:** Socio-demographic characteristics of the study participants

Variable	Category	N=207 n (percentage) or Mean ± SD
**Age**		22.7 ± 14.3
**Sex of the victim**	Male	134 (64.7)
	Female	73 (35.3)
**Occupation**	Student	47 (23.2)
	Child	46 (22.7)
	Housewife	14 (6.9)
	Peasant	23 (11.3)
	Business	40 (19.70)
	Others^1^	33 (16.4)
**District**	Kampala	118 (60.8)
	Wakiso	54 (27.8)
	Others^2^	22 (11.3)

1 Motor cyclist, taxi conductor, driver, teacher, secretary, farmer, cleaner, chef, guard, welder, and mason.

2 Mukono, Luwero, Kayunga, Mpigi, Mubende, Kiboga, Rukungiri

**Table 2:** Clinical characteristics of the study participants

Variable	Category	N=207 n (percentage) or Mean ± SD
Body part	Lower limb	149 (72.0)
	Upper limb	49 (23.7)
	Others^3^	9 (4.3)
Time of animal bite	Day time	167 (80.7)
	Night time	40 (19.3)
Time to care in Mulago (Days )		2.6 ±4.3
First bite	Yes	200 (96.6)
	No	7 (3.4)
First aid offered	Yes	50 (24.2)
	No	157 (75.8)
Where first aid was offered (N=50)	Clinic	41 (82.0)
	Hospital	7 (14.0)
	Home	1 (2.0)
	By stander	1 (2.0)
Victim’s activity	Playing with dog	41 (19.8)
	Beating dog	14 (6.8)
	Walking/running	152 (73.4)
Treatment given at Mulago Hospital	Anti-rabies	205 (99.0)
	Wound dressing	2 (1.0)

Others^3^ represents finger, neck, hips, foot, mouth, scrotum, thigh, face, left

**Table 3:** Characteristics of the animals

Variable	Category	N=207 n (Percentage)
**Animal bite injury**	Dog	197 (95.2)
	Cat	7 (3.4)
	Other (e.g.fox)	3 (1.4)
**Dog owner**	Home	24(11.6)
	Neighbor	73(35.3)
	Unknown	109 (52.7)
	Wild	1(0.5)
**Rabies immunization status of the dog**	Immunized	23(11.1)
	Not immunized	60(29.0)
	Unknown	124 (57.9)
**Dogs under restraint/chained**	Yes	3 (1.4)
	No	204 (98.6)
**Dog signaled /ordered**	Yes	3 (1.4)
	No	203(98.1)
	unknown	1 (0.5)
**Dogs that attacked the victim**	Yes	93(45.2)
	Dead	16(7.8)
	Unknown	97(47.1)
	Killed by someone	11 (73.3)
	Died by itself	4 (26.7)

**Table 4:** Results of regression analysis of the factors associated with animal bites at Mulago Hospital

Variable	Categories	cIRR	95% CI	P values	aIRR IRR	95% CI	P value
**Age**		1.0	1.0-1.01	0.004			
**First aid given**	No	2.1	1.8-2.5	0.00	1.7	1.4-2.1	0.00
**Body part**	Others	1					
	Lower limbs	1.7	1.0-2.9	0.07			
	Upper limbs	1.3	0.7-2.3	0.4			
**Number of dogs**		0.7	0.5-1.0	0.05	0.8	0.6-1.1	0.24
**Time of day**	Morning	1			1		
	Day	1.8	1.4-2.3	0.00	1.6	1.3-2.1	0.00
	Evening	1.6	1.2-2.0	0.00	1.3	1.0-1.7	0.05
	Night	1.0	0.7-1.3	1.0	1.0	0.8-1.4	0.87
**Dog owner**	Home	1			1		
	Neighbor	0.6	0.5-0.8	0.00	0.7	0.6-1.0	0.03
	Unknown	0.5	0.4-0.6	0.00	0.7	0.5-0.9	0.01
	Wild	0.2	0.03-1.6	0.14	0.2	0.03-1.4	0.1
**Previous bite**		0.6	0.3-1.1	0.104	0.7	0.6-1.1	0.24
**Previous treatment**	Yes	1			1		
	No	5.4	3.9-7.5	0.00	4.8	3.5-6.8	0.00
	Unknown	0.8	0.6-1.0	0.05	1.0	0.7-1.4	0.96
**Animal**	Dog	1					
	Cat	4.0	3.0-5.4	0.00			
	Fox	0.2	0.03-1.5	0.12			
**District**	Others	1			1		
	Kampala	0.6	0.5-0.7	0.00	0.6	0.5-0.8	0.00
	Wakiso	0.9	0.7-1.1	0.18	0.8	0.6-1.0	

**cIRR**- crude incident risk ratio, aIRR- adjusted incident risk ratio

### Clinical characteristics of the study participants

The majority of the victims 199 (96.1%) were bitten by a lone unrestrained and un-signaled dog that had bitten someone else in 22.2% of cases, and eight victims (0.04%) were attacked in canine gangs of 2-5 dogs. The victims were mainly bitten during the day time, in which 101 victims (96.6%) sustained extremity injuries mostly on the lower limbs in 149 (71.6%). Nearly all the victims of animal bites 200 (97.1%) reported this as the first bite. One hundred and eighty nine victims (91.3%) presented within 2.6 days (SD ± 4.3) of the dog bites, with only 50 (24.5%) reporting to have received first aid services especially in clinics 41 (74.6%). All injuries were minor and managed on out-patient basis with wound dressing, analgesics, prophylactic antibiotics and anti-rabies vaccination. Table 2 shows the clinical characteristics of the study participants.

### Characteristics of the animals

Regarding the characteristics of the animals, 197 (95.6%) were dogs, 7 (3.3%) were cats and 3 (1.5%) were foxes. Only 23 (11.1%) were reportedly vaccinated against rabies. Home or neighborhood dogs were more likely to be vaccinated and they injured victims who were either playing or beating them, while the wild dogs whose vaccination history was unknown, injured victims who were walking or running. The dog bite victims were mostly strangers or neighbours. More than half of the victims 109 (52.7%) were injured by dogs whose ownership was unknown, commonly in reference to stray or wild dogs.

### Results of multi-variate analysis of the factors associated with animal bites

In multivariate analysis in Table 4, the victims who received prior first aid had a rate of 1.7 times greater for seeking treatment at Mulago hospital (IRR 1.7, 95% CI 1.4-2.1) compared to those that had no prior first aid. Participants who sustained the animal bite injuries during day time had a rate of 1.6 times greater for seeking treatment at Mulago hospital (IRR 1.6, 95% CI 1.3-2.1) compared to those that sustained injuries at other times. Participants bitten by unknown dog and participants residing in Kampala had IRR 0.7, 95% CI 0.5-0.9 and IRR 0.6, 95% CI 0.5-0.8 respectively of accessing treatment at Mulago hospital compared to bitten by known dog and not residing in Kampala.

## Discussion

We found a prevalence of animal bite injuries of 0.8% among victims presenting to the national referral hospital's accident and emergency unit and nearly all were due to dog bites. This was in agreement with a prevalence of 0.87% reported by Kelly Auma *et al.* in Kakamega, Kenya [7] but much lower than 2.8% reported by Ogendi and Ayisi, in a provincial general hospital in Nyanza, Western Kenya [8]. Kampala, where majority of the dog bite victims in our study reside is home to over 20,000 dogs with a dog population density of about 105/Km^2^ which is way higher than Kisumu in Nyanza province with an average dog population density of 49 dogs per Km^2^. Other researchers have also found low prevalence [9] of animal bite injuries or low incidence [10, 11] of dog bite injuries and most of the animals are not vaccinated against rabies [3, 10, 12]. One would therefore expect a higher prevalence of dog bite injuries in our study compared to Nyanza, Western Kenya. In as far as the age of the injury victims is concerned multiple studies report that younger people are more at risk of dog bites than older people [1-5, 13-18]. The percentage of dog bite victims aged under 18 years (40%) in our study was similar to that in Nyanza, Kenya, and therefore does not explain the high prevalence of dog bites in Nyanza, Western Kenya. In our study, most of the injury victims sought first aid from clinics in the city and only sought anti-rabies vaccine from the study site. It is possible that the victims could not purchase the anti-rabies vaccine at the private health units that stock it because it was expensive or because the lower level public health facilities are not stocked with anti-rabies vaccine by central authorities in Uganda [1, 3]. The victims' and public's concern over animal bite injuries is the potential risk of contracting the deadly zoonotic disease rabies [3, 4, 7, 12]. This is what worries the victims and compels them to seek medical attention in a hospital setting, expecting post exposure prophylaxis against rabies [3, 4, 7, 12]. Nyanza province in Kenya has a comprehensive rabies prevention and control program that involves raising awareness about dog bites and rabies. This might be responsible for a high number of dog bite victims reporting to the general provincial hospital in order to access care and anti-rabies vaccine which is not routinely supplied by the Kenyan government to public hospitals and hence a high prevalence of dog bite injuries is registered.

The majority of the dog bite injury victims in our study were male or children with a male to female ratio of nearly 2:1, in agreement with multiple studies [7, 10, 17]. This could perhaps be as a result of men and boys being more curious and more likely than their female counterparts to provoke the canines they encounter in their environments which therefore bite them in self-defense. Dog bite injuries reportedly decrease with increasing male age [17]. Most of the victims in our study were running when they encountered the dogs that bit them. Multiple studies also report children to be a high risk group for dog bites because of their risk-taking behaviour such as playing with or beating the dogs as well as failure to identify stress postures of dogs that are likely to bite them [13-15]. Regarding the characteristics of the dog bite injuries, our study corroborates the observation that most animal bite injuries involve the extremities [3, 10]. Other researchers have also found low prevalence [9] of animal bite injuries or low incidence [10, 11] of dog bite injuries. Majority of the bite injuries in our study involved the lower limbs. They were minor injuries that did not require admission in the hospital in contrast to a study in Kisumu, Western Kenya that reported four (4) victims that required admission [8]. Like what has been observed elsewhere, majority of the animal bite injuries were due to dog bites [1-18]. More than half (52%) of the dogs in our study were unknown to the victims in agreement with other studies [3, 16] and therefore their rabies immunization status was unknown. These unknown dogs that were not signaled or ordered bit their victims while they walked or ran in contrast to home or neighbourhood dogs that were likely to be vaccinated and they injured victims who were either playing or beating them. Almost half of the animal bites in our study involved dogs kept as pets by the family or neighbor. This observation is in agreement with findings by other researchers [4, 16, 17]. This pattern of attack is in agreement with multiple studies that have looked at strategies to prevent and control dog bites and rabies [1-4, 7, 12, 17]. Most of the bites occurred during day time, to the lower limbs, and the victims were mainly strangers, neighbours and most did not get any first aid prior to reporting to a health facility or Mulago Hospital. Lack of information in communities about dog ownership and their immunization status poses a grave probability of rabies disease persistence, and the likelihood of continued dog-to-human transmission.

In our study, 4 dogs died by themselves. Furthermore, although our study did not involve follow up of the victims, it is known that animal bite injuries have the risk for not only rabies transmission but also infection and cost implications for people affected [4-6]. The victims sought care on average 2.6 days (SD 4.3) after the bite injuries similar to a study in which victims in 10 health centres in Uganda presented with a median of 2 days. Participants that received prior first aid from clinics and those that sustained the animal bite injuries during the day, were more likely to seek anti-rabies vaccine at Mulago hospital early on after the injury. The likely explanation may be that the health personnel that gave the first aid sensitized them about the rabies and stressed the need to urgently access the anti-rabies vaccination at Mulago hospital. Accessing transport to Mulago hospital during the day in case of a bite injury is easier than during the night time. Animal bite injury victims residing in Kampala were less likely to present early to Mulago hospital which was rather surprising. Probably prior experience and bias about frequent drug stock outs at Mulago hospital which is a public health facility in Kampala deterred them from presenting early as they first sought anti-rabies vaccine from private clinics or low-level health facilities. The preference for consulting practitioners outside mainstream medicine may be more marked in Kampala than the neighbouring districts and upcountry.

## Conclusion

Dog bites injuries from unrestrained, un-signaled dogs are the commonest source of animal bite injuries especially among men and children < 18 years. The rabies vaccination status of the dogs was only confirmed for a very small number of dogs, as majority were unknown and likely stray dogs. Government is urgently required to set up an intervention team to confront the problem of dog bites by especially unknown dogs in order to prevent a grave probability of a looming canine rabies epidemic.

### What is known about this topic

- Animal bites are a public health problem;
- Most animal bites occur among the rural poor;
- Dogs responsible for the majority of animal bites.

### What this study adds

- Most of the bites occurred within Kampala city and its neighbourhoods;
- The majority of the offending animals in Kampala city were wild (or owner unknown to the victim), marauding, unrestrained and un-signaled dogs which had no rabies vaccination or whose rabies vaccination status was unknown to the victims;
- Anti-rabies vaccine for dog bite victims is mostly not available in lower level health facilities outside Kampala city.

## Competing interests

The authors declare no competing interests.

## References

[1] World Health Organization (2018). Animal bites.

[2] Meslin FX, Briggs D (2013). Eliminating canine rabies, the principal source of human infection: what will it take?. Antiviral Res.

[3] Fèvre EM, Kaboyo RW, Persson V, Edelsten M, Coleman PG, Cleaveland S (2005). The epidemiology of animal bite injuries in Uganda and projections of the burden of rabies. Trop Med Int Health.

[4] Presutti RJ (2001). Prevention and treatment of dog bites. Am Fam Physician.

[5] Anderson CR (1992). Animal bites: guidelines to current management. Postgrad Med.

[6] Benson LS, Edwards SL, Schiff AP, Williams CS, Visotsky JL (2006). Dog and cat bites to the hand: treatment and cost assessment. J Hand Surg Am.

[7] Kelly AN (2016). Vertebrate animal bite/scratch injuries and management among patients reporting at Kakamega Provincial General Hospital.

[8] Ogendi JO, Ayisi JG (2011). Causes of injuries resulting in a visit to the emergency department of a Provincial General Hospital, Nyanza, western Kenya. Afr Health Sci.

[9] Odero WO, Kibosia JC (1995). Incidence and characteristics of injuries in Eldoret, Kenya. East Afr Med J.

[10] Aghahowa SE, Ogbevoen RN (2010). Incidence of dog bite and anti-rabies vaccine utilization in the, University of Benin Teaching Hospital, Benin city, Nigeria: a 12-year assessment. Vaccine.

[11] Abubakar SA, Bakari AG (2012). Incidence of dog bite injuries and clinical rabies in a tertiary health care institution: a 10-year retrospective study. Ann Afr Med.

[12] Jovita M (2005). Do not ignore a dog bite.

[13] Bernerdo LM, Gardner MJ, Rosenfield RL, Cohen B, Pitetti R (2002). A comparison of dog bite injuries in younger and older children treated in a paediatric emergency department. Paediatr Emerg Care.

[14] Schalamon J1, Ainoedhofer H, Singer G, Petnehazy T, Mayr J, Kiss K, Höllwarth ME (2006). Analysis of dogbites in children who are younger than 17 years. Pediatrics.

[15] Weiss HB, Friedman DI, Cohen JH (1998). Incidence of dogbite injuries treated in emergency departments. JAMA.

[16] Ahmed H, Chafe UM, Magaji AA, Abdul-Qadir A (2000). Rabies and dog bite in children: a decade of experience in Sokoto Nigeria. Sokoto Journal of Veterinary Sciences.

[17] Gilchrist J, Sacks JJ, White D, Kresnow MJ (2008). Dog bites: still a problem?. Inj Prev.

[18] Lewis KT, Stiles M (1995). Management of cat and dog bites. Am Fam Physician.

